# Xenos peckii vision inspires an ultrathin digital camera

**DOI:** 10.1038/s41377-018-0081-2

**Published:** 2018-10-24

**Authors:** Dongmin Keum, Kyung-Won Jang, Daniel S. Jeon, Charles S. H. Hwang, Elke K. Buschbeck, Min H. Kim, Ki-Hun Jeong

**Affiliations:** 1 Department of Bio and Brain Engineering, Korea Advanced Institute of Science and Technology (KAIST), 291 Daehak-ro, Yuseong-gu, Daejeon, 34141 Korea; 2 School of Computing, Korea Advanced Institute of Science and Technology (KAIST), 291 Daehak-ro, Yuseong-gu, Daejeon, 34141 Korea; 3 Department of Biological Sciences, University of Cincinnati, Cincinnati, OH 45221-0006 USA

## Abstract

Increased demand for compact devices leads to rapid development of miniaturized digital cameras. However, conventional camera modules contain multiple lenses along the optical axis to compensate for optical aberrations that introduce technical challenges in reducing the total thickness of the camera module. Here, we report an ultrathin digital camera inspired by the vision principle of *Xenos peckii*, an endoparasite of paper wasps. The male *Xenos peckii* has an unusual visual system that exhibits distinct benefits for high resolution and high sensitivity, unlike the compound eyes found in most insects and some crustaceans. The biologically inspired camera features a sandwiched configuration of concave microprisms, microlenses, and pinhole arrays on a flat image sensor. The camera shows a field-of-view (FOV) of 68 degrees with a diameter of 3.4 mm and a total track length of 1.4 mm. The biologically inspired camera offers a new opportunity for developing ultrathin cameras in medical, industrial, and military fields.

## Introduction

Design diversity of natural compound eyes is a compelling source of biological inspiration for compact optical imaging systems. The facetted nature is particularly beneficial because multiple apertures and tiny lenses can transfer a similar amount of visual information as a single large lens^1,2^. The unique configuration further exhibits intriguing optical functions, such as a large FOV, high sensitivity to motion, and minimal off-axis aberration^3,4^. In particular, the anatomical features and the imaging principle of the *Xenos peckii* eye are very different from typical compound eyes. The individual optical unit consists of a relatively large convex facet lens and multiple photoreceptor cells, whereas that of the apposition compound eye has one to several photoreceptor cells in a single unit. Each optical unit, called an eyelet, detects part of the overall FOV, with improved spatial resolution and sensitivity compared to other compound eyes^5–7^.

Conventional microfabrication technology mostly restricts the implementation of artificial compound eyes (ACEs) to a planar configuration, which comprises microlenses and light blocking structures such as pinhole arrays or signal separator layers^8–12^. A planar configuration still has substantial limitations to the total FOV of the camera because each microcamera, which consists of a single lens and photodetector, has constraints in tilting the viewing angle. In contrast, spherically shaped ACEs can provide large FOVs similar to natural compound eyes; however, they still require additional optical components to relay the received visual signals to the photodetector due to the curved image plane^13–16^. As an alternative, prisms or freeform microlens arrays can be utilized to tilt the principal optical axis of each channel to secure both an enlarged FOV and a flat image plane^17–20^. Recently, hemispherical shapes of photodetector arrays were combined with a spherical ACE, but the manufacturing methods are still not yet fully compatible with conventional semiconductor fabrication methods^21,22^.

**Fig. 1:**
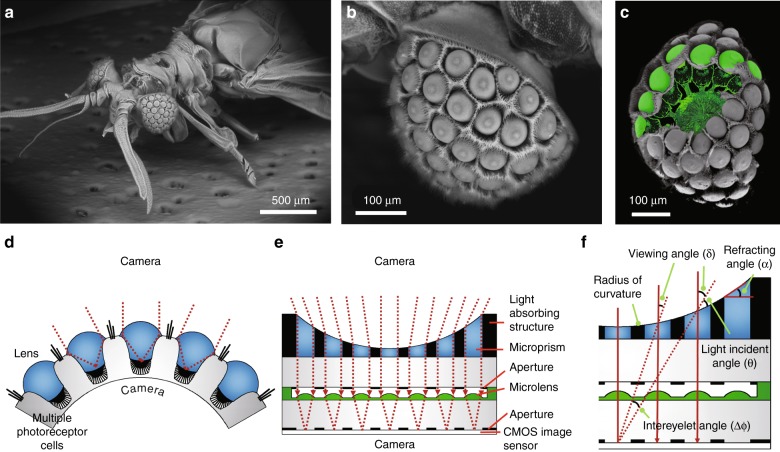
Natural Xenos peckii eye and the biological inspiration for the ultrathin digital camera. Scanning electron micrographs of **a** Xenos peckii, an endoparasite of paper wasps, **b** Xenos peckii eye, and **c** superimposed fluorescently stained eyelets. Schematic diagrams of **d** a single eye of Xenos peckii and **e** an ultrathin digital camera inspired by its vision principle. Unlike the eyes of other arthropods, each eyelet of Xenos peckii consists of a relatively large lens and multiple photoreceptor cells, which resolve a partial image within the total FOV. The bioinspired camera comprises concave microprisms, microlenses, and apertures on a CIS. **f** Design parameters of the bioinspired camera

Here, we report an ultrathin digital camera inspired by the vision principle of *Xenos peckii*, an endoparasite of paper wasps. Figure 1a, b are scanning electron microscope (SEM) images of an adult male *Xenos peckii* and one single eye, respectively. Each eyelet of *Xenos peckii* has more than one hundred photoreceptor cells. Figure 1c shows fluorescently stained optical units (called eyelets) superimposed onto an SEM image of a *Xenos peckii* eye, which illustrates in detail a relatively large lens and underlying receptor arrays. Each eyelet detects a partial image within the total FOV (Fig. 1d), thus improving the spatial resolution and the sensitivity^5–7^. Figure 1e shows a schematic illustration of a biologically inspired ultrathin digital camera. An artificial eyelet (channel) features a single microprism to tilt the optical axis, a single microlens to focus light from the microprism, and an aperture to block light from adjacent channels. The individual channel is surrounded by a light-absorbing medium to reduce the optical crosstalk between neighboring channels. A viewing angle can be defined as the angle between the center axis of the channel and the incident ray (Fig. 1f). The intereyelet angle (*Δϕ*), i.e., the angular difference between the viewing angles of each channel, can be obtained as *Δϕ* = (*θ*_1_ + *α*_1_) *−* (*θ*_2_ + *α*_2_), where *θ* is a light incident angle, *α* is a refracting angle of the microprism, and the numbers represent two neighboring channels. The FOV (Δ*φ*) of a single eyelet is determined by Δ*φ* = *2 arctan* (*d/*2*f*), where *f* and *d* are the focal length of the microlens and the diameter of the bottom aperture, respectively. The image overlap angle between neighboring channels can be further utilized to improve the image resolution during the image reconstruction process. The polychromatic modulation transfer function (MTF) value was also analyzed by ray tracing and slightly decreased as the position of the channel became farther from the center of the camera (Supplementary Figure S1).

## Results and discussion

**Fig. 2:**
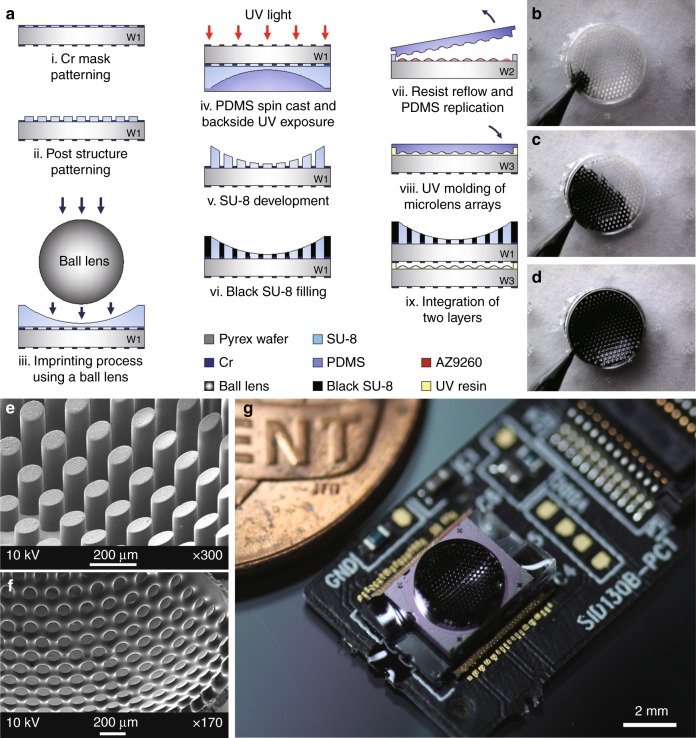
Fabrication methods and features of the bioinspired ultrathin digital camera. **a** Microfabrication steps. The microprism plate was formed by using ball lens imprinting and backside lithography. i Cr mask patterning on both sides of the substrate, ii micropost arrays patterning, iii SU-8 imprinting using a ball lens, iv PDMS spin cast and UV exposure from the backside of the glass substrate, v SU-8 development, vi 3D capillary filling of the black SU-8, vii the microlens plate was separately fabricated by the thermal reflow process; PDMS casting of the microlens arrays, viii UV molding of the PDMS replica, and ix camera assembly. The microprism and microlens plates were precisely assembled on an image sensor. **b**–**d** Optical images of 3D capillary filling of black SU-8. Black SU-8 monomer spreads between the microprisms during microdispensing. **e**, **f** SEM images of concave microprism arrays before and after filling with black polymer. **g** Optical image of the fully assembled ultrathin digital camera

Figure 2 illustrates the microfabrication procedure of the biologically inspired ultrathin digital camera. Honeycomb-packed concave hexagonal microprism and microlens plates are separately fabricated and integrated together on a conventional CMOS image sensor (CIS). First, a concave microprism plate was fabricated by combining ball lens imprinting and backside lithography. Thin chrome layers were precisely defined on both sides of a glass substrate by using a lift-off process as a photomask and an aperture. Then, 30 μm thick micropost arrays of SU-8 2025 were photolithographically defined by using backside ultraviolet (UV) exposure. Subsequently, SU-8 2150 was spin-coated and soft-baked on the micropost arrays and then pressed by a fluorocarbon-coated ball lens. During an additional soft-bake process, gravitational pulling causes the ball lens to move downward and causes the soft-baked SU-8 film to form a concave shape. Note that the micropost arrays support the ball lens at the center region. Polydimethylsiloxane (PDMS) was then spin-casted on the SU-8 film and cured to maintain the concave shape during the post-exposure bake (PEB) process. UV light was exposed from the backside with the embedded Cr photomask. The PDMS slab was peeled off after the PEB process, and the concave microprism arrays were clearly defined during the development process. 3D capillary filling of black SU-8 (Gersteltec, GMC 1040) was performed to reduce the optical crosstalk between the microprisms (Fig. 2b–d, see also Supplementary Video SV1). Second, the microlens plate was separately prepared by using a resist reflow process of AZ 9260 photoresist after patterning SU-8 spacers, which prevent direct contact between the microlens arrays and the substrate. The microlens arrays were replicated with PDMS at room temperature and further transferred to UV curable optical resin (Norland optical adhesive, NOA 61) on a glass substrate. Finally, the microprism and microlens plates were assembled with optical alignment. The optical alignment between the microprism and the microlens plates was precisely performed by matching the predetermined alignment keys on each substrate: the microlens plate was placed and rotated on the rotation stage and the microprism plate was translated on the lateral direction above the microlens plate by using the translational stage. Both substrates were permanently glued after optical alignment with a UV curable polymer. Figure 2e, f show the SEM images of the microprism arrays before and after filling with black SU-8. The diameter and the gap between the channels are 100 μm and 80 μm, respectively. After microassembly of the microprism and microlens plates, the device was fully integrated on a CIS (2M pixels, unit pixel: 1.75 μm x 1.75 μm), and the total track length of the bioinspired ultrathin digital camera is 1.4 mm (Fig. 2g).

**Fig. 3:**
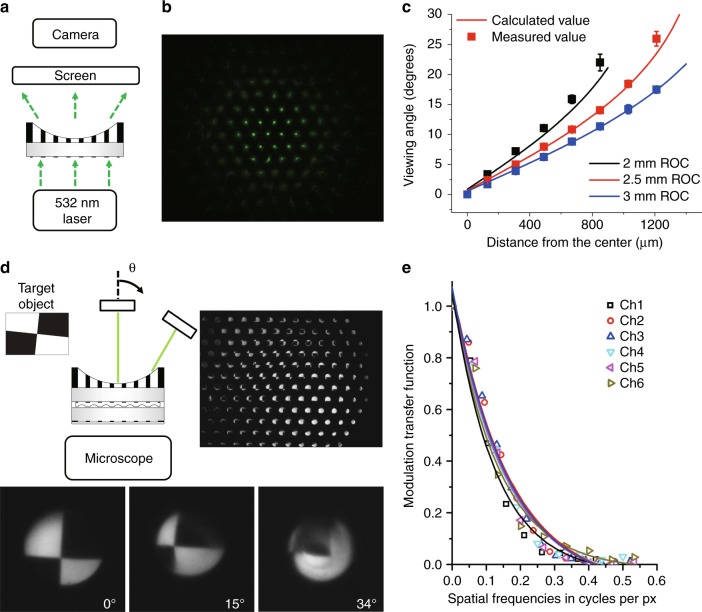
Viewing angle and modulation transfer function. **a** Schematic illustration of the experimental setup for the viewing angle measurement, **b** light spots imaged on a screen, **c** the measured and calculated viewing angles for the concave surfaces of 2, 2.5, and 3 mm in the ROC (*n* = 4, std < 1.2 degree). The large ROC leads to a small FOV of the microprism plate. **d** Schematic representation of an experimental setup for measuring the FOV, slanted edge target, and magnified captured images at three different incident angles. **e** MTF for the individual channels. The MTFs show uniform optical performance in the individual channels. The off-axis aberration is negligible up to channel 6

The viewing angle was measured by using the experimental setup illustrated in Fig. 3a. The concave microprism plate was illuminated from the backside using a collimated 532 nm laser beam, and the steered beam spots were captured on a screen (Fig. 3b). Figure 3c compares the trigonometrically calculated and measured viewing angles for the concave microprism plate of 2, 2.5, and 3 mm in the radius-of-curvature (ROC). The measured average viewing angles match well with the calculated values. The large ROC clearly decreases the total FOV. For the 2.5 mm ROC, the designed FOV of a single eyelet is 19.1 degrees, and the intereyelet angle varies from 2.5 degrees to 5.3 degrees. Consequently, the designed image overlap angle ranges from 13.8 degrees to 16.6 degrees (Supplementary Figure S2). Before integration with the CIS, the total FOV was also measured by rotating a target object in front of the concave microprism plate. The corresponding images were captured from three different incident angles for different channels (Fig. 3d). The visual input signals for a target object were measured up to an incident angle of 34 degrees, which also indicates 68 degrees of the total FOV (Supplementary Figure S3). Figure 3e shows the MTF for six different channels. The MTFs were measured from a slanted edge target at six different angles of incidence, each of which corresponds to the on-axis direction of the channels. The experimental results show that the MTFs are uniform among the individual channels. Note that the images in Fig. 3d and the images used to calculate MTF in Fig. 3e are different due to the different microscopic magnifications for the FOV calculation and MTF measurement.

**Fig. 4:**
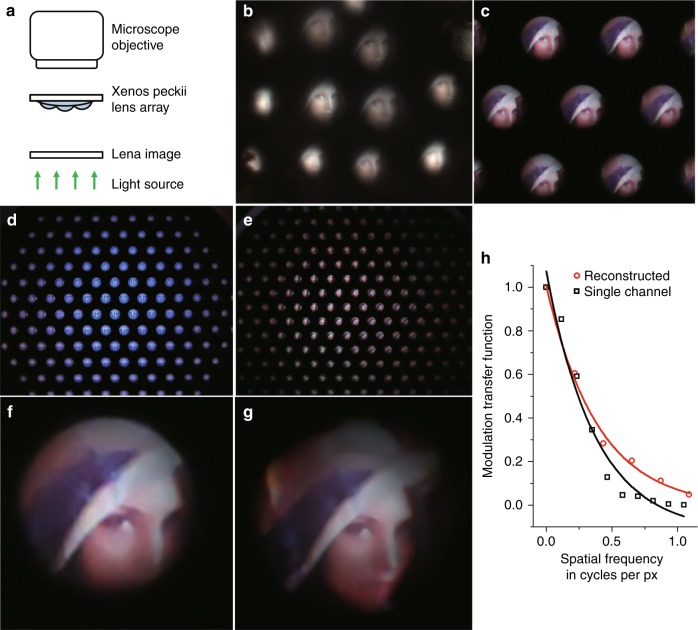
Optical images captured by the bioinspired ultrathin digital camera. **a** Experimental setup for capturing the optical images through a natural *Xenos peckii* eye. The “Lena” standard test image was printed out on the paper and back-illuminated through an optical microscope. **b** Optical image captured from a dissected natural *Xenos peckii* eye. **c** Captured optical image from the biologically inspired digital camera with Lena displayed on a laptop monitor. Each channel of the camera receives visual signals from a slightly different direction, similar to that of the natural eye. **d**, **e** ‘KAIST**’** and Lena images captured on the total active pixel area of the CIS. **f** Captured image from a single channel on center. **g** Reconstructed image from 19 channels. **h** Comparison of MTFs between the single channel image and the reconstructed image. The MTF50 is 154 cycles∙mm^-1^ in the single channel image, which was increased up to 181 cycles∙mm^-1^ after combining the captured images from 19 channels

Figure 4a represents the experimental setup for capturing optical images through a dissected natural *Xenos peckii* eye. The target “Lena” image was printed onto a paper and back-illuminated through an upright microscope, and the *Xenos peckii* lens arrays were hung by surface tension on the bottom of a slide glass in physiological saline solution (see details in methods section). Figure 4b shows the partial images from each eyelet of *Xenos peckii*, which perceives slightly different views of the Lena image. Figure 4c also shows the captured image from the biologically inspired camera after the CIS integration. In this case, the Lena image was displayed on a laptop monitor. The individual camera channels detect slightly different parts of the full FOV, such as the natural eyelets. Figure 4d, e demonstrate the captured image of the ‘KAIST’ and Lena on the total active pixel area of the CIS. Finally, a single image with high resolution was reconstructed from the partial images in Fig. 4f (see details in methods section). Figure 4g, h show the reconstructed image obtained from the 19channels and the MTFs between the single channel and reconstructed images, respectively. The total FOV of the reconstructed image was larger than that of a single channel image by 30%. The calculated MTF50 was 289 cycles∙mm^**−1**^, whereas the measured MTF50 was 154 cycles∙mm^**−1**^ (0.263 cycles∙pixels^**−1**^) in the single channel image. The measured value was increased up to 181 cycles∙mm^**−1**^ (0.308 cycles∙pixels^**−1**^) in the reconstructed image. The MTFs of the reconstructed image were significantly increased in high frequency compared to that of a single channel, which clearly indicates that the image resolution is improved by increasing the edge sharpness. The difference between the calculated and the measured values can be explained by the following reasons: the Nyquist frequency of the CIS, i.e., one half of the reciprocal of the pixel pitch, limits the spatial sampling rate of the optical imaging system^23^. The fabrication tolerance, such as the residual thickness under the microlens arrays, the ROC of the microlenses, or the optical alignment could also result in a decrease in the MTF. In addition, the multiple diaphragm layers of thin chrome film used to reduce the optical crosstalk between adjacent channels could further cause image degradation due to the reflectivity of approximately 40%. This problem can be improved by employing an antireflection coating on both sides of the metal layers or black chromium (BCr) instead^24^.

In summary, this work has successfully demonstrated a fully packaged ultrathin digital camera inspired by the vision principle of *Xenos peckii*. The biologically inspired camera comprises two stacked plates of curved microprism arrays and planar microlens arrays on a conventional CIS. The curved microprism plate was fabricated by using ball lens imprinting and backside lithography with an embedded metal mask. Optical crosstalk between the microprism channels was blocked by using 3D capillary filling with SU-8 black polymer. The camera shows the FOV of 68 degrees with a diameter of 3.4 mm and a total track length of 1.4 mm. The imaging performance has been successfully demonstrated, and the final image has been reconstructed from the captured image of each channel. This ultrathin digital camera provides a new direction for developing compact imaging systems for surveillance and reconnaissance instruments, medical imaging apparatuses, mobile devices, and other optical sensors.

## Materials and methods

### Experimental setup for imaging with a *Xenos peckii* eye

To obtain the Lena images through the lens arrays of *Xenos peckii*, insects were first anesthetized by cooling and were then decapitated, and the lens arrays of one of the eyes were removed. The inner surfaces of the arrays were gently cleaned from residual pigment and other cellular debris with a soft brush. Thereafter, it was placed on top of a small droplet of physiological saline solution that was placed on a microscopy cover slip^25^. Following the hanging-drop method, the cover-slip was inverted in such a way that the lens arrays, held by surface tension, floated to the bottom of the droplet^5,26^. The arrays were placed on the stage of a compound microscope (Olympus BX 51) from which the condenser was removed. A printout of the Lena image (on photographic paper) was placed below the microscope stage, within the light path of the microscope’s bottom illumination. In this way, the Lena print was in the light path of the lens arrays, which resulted in a series of small images (one for each *Xenos* lens) that could then be visualized through the microscope and imaged with a digital camera (Retiga 2000R, Qimaging).

### 3D capillary filling of Black SU-8

Black SU-8 was precisely dispensed on the edge of the microprism arrays by using a glass micropipette mounted on an xyz micromanipulator. The black SU-8 spreads through the interstitial gaps of the microprism post arrays due to capillary forces. The flow rate of the black SU-8 through the glass micropipette was precisely controlled with custom-made pumping system. After 3D filling, the black SU-8 was soft-baked at 95 °C on a hot plate. The top side of the device was then exposed to UV light, followed by post-exposure bake.

### Image reconstruction algorithm

Each microprism image was initially registered to the reference image by shifting images to seek the minimum normalized cross-correlation values between images. The registered multiple microprism images were then used to reconstruct a higher resolution image using a super-resolution approach^27^. We built a cost function to reconstruct a higher resolution image$X$ from multiple low-resolution input images$Y$:

1 $$\mathop {{\min }}\limits_X \mathop {\sum }\limits_{k = 1}^N \left\| {F_kX - Y_k} \right\|_2^2 + {\mathrm{\lambda \Gamma }}\left( X \right)$$

where $F_k$ is a geometric motion operator between $X$ and the *k-*th image $Y_k$ and ${\mathrm{\lambda }}$ is a regularization parameter. This optimization problem was solved with a bilateral-TVL1 regularizer ${\mathrm{\Gamma }}(X)$:

2 $${\mathrm{\Gamma }}\left( {\mathrm{X}} \right) = \mathop {\sum }\limits_{l = 0}^P \mathop {\sum }\limits_{m = 0}^P \alpha ^{\left| m \right| + \left| l \right|}\left\| {X - S_x^lS_y^mX} \right\|_1^1$$

where $\alpha$ is a scale factor and $S_x^l\,{\mathrm{and}}\,S_y^m$ are the shift operators along the horizontal and vertical axes, respectively. The objective function was solved using the conjugate gradient descent method with parameters *λ* = 2 and *α* = 0.6.

## Electronic supplementary material

supplementary information

3D capillary filling of black SU-8
