# Supplementary material for: Xenos peckii vision inspires an ultrathin digital camera
Source: Light Sci Appl. 2018 Oct 24;7:80. doi: 10.1038/s41377-018-0081-2 (PMC6199290; doi:10.1038/s41377-018-0081-2)
Supplement: Supplementary file 1 — supplementary information [file 41377_2018_81_MOESM1_ESM.pdf]

Supplementary Information

Xenos peckii vision inspires an ultrathin digital camera

Dongmin Keum[1], Kyung-Won Jang[1], Daniel S. Jeon[2], Charles S. H. Hwang[1], Elke K. Buschbeck[3], Min H. Kim[2], Ki-Hun Jeong[1]*

[1]*Department of Bio and Brain Engineering, Korea Advanced Institute of Science and Technology (KAIST), 291 Daehak-ro, Yuseong-gu, Daejeon 34141, Korea*

[2]*School of Computing, Korea Advanced Institute of Science and Technology (KAIST), 291 Daehak-ro, Yuseong-gu, Daejeon 34141, Korea*

[3]*Department of Biological Sciences, University of Cincinnati, Cincinnati, OH 45221-0006, USA*

*email: kjeong@kaist.ac.kr

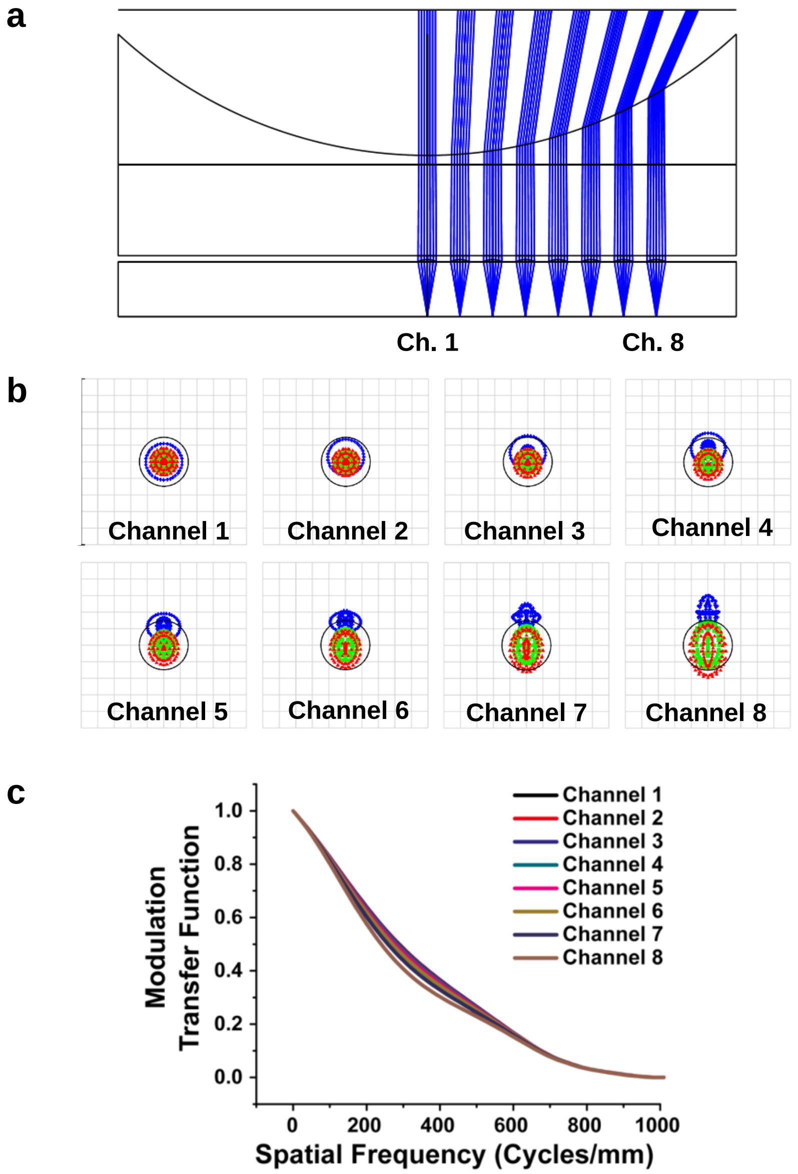

Supplementary Figure S1 | Simulated optical performances of the *Xenos peckii* vision inspired ultrathin digital camera. (a) Optical layout of the camera. **(b)** Root mean square (RMS) radius of spot diagram with Airy disc of 8 channels. The channel number increases from the center to the edge region. The radius of curvature (ROC) and the thickness of microlens arrays were optimized with the RMS spot radius. The blue spots, green spots, and red spots represent the wavelengths of 486 nm, 588 nm and 656 nm, respectively. The chromatic aberration becomes apparent as the incident angle increases, however the spots do not deviate much far from the Airy disc. **(c)** Polychromatic modulation transfer function (MTF)

of 8 channels for the concave surface of 2.5 mm radius of curvature. The MTF values slightly decrease as the channel number increases.

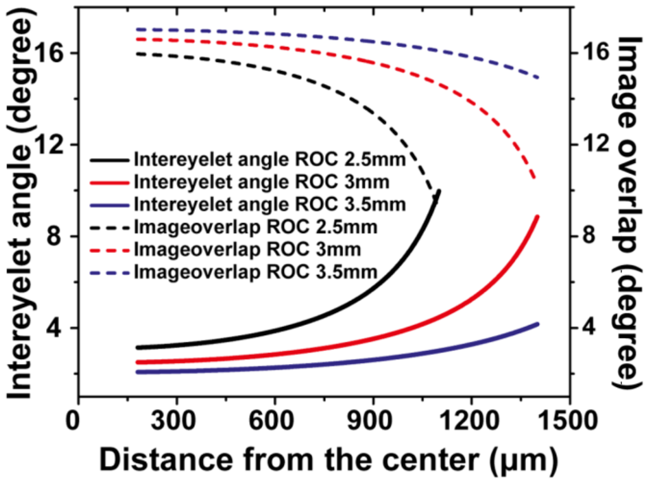

Supplementary Figure S2 | Relation between the intereyelet angle and the image overlap. Total field-of-view (FOV) of the camera and the image overlap between the channels are in trade-off relationship. The larger the ROC of concave surface, the larger the image overlap between the channels. However, the large ROC leads to the small total FOV of the camera.

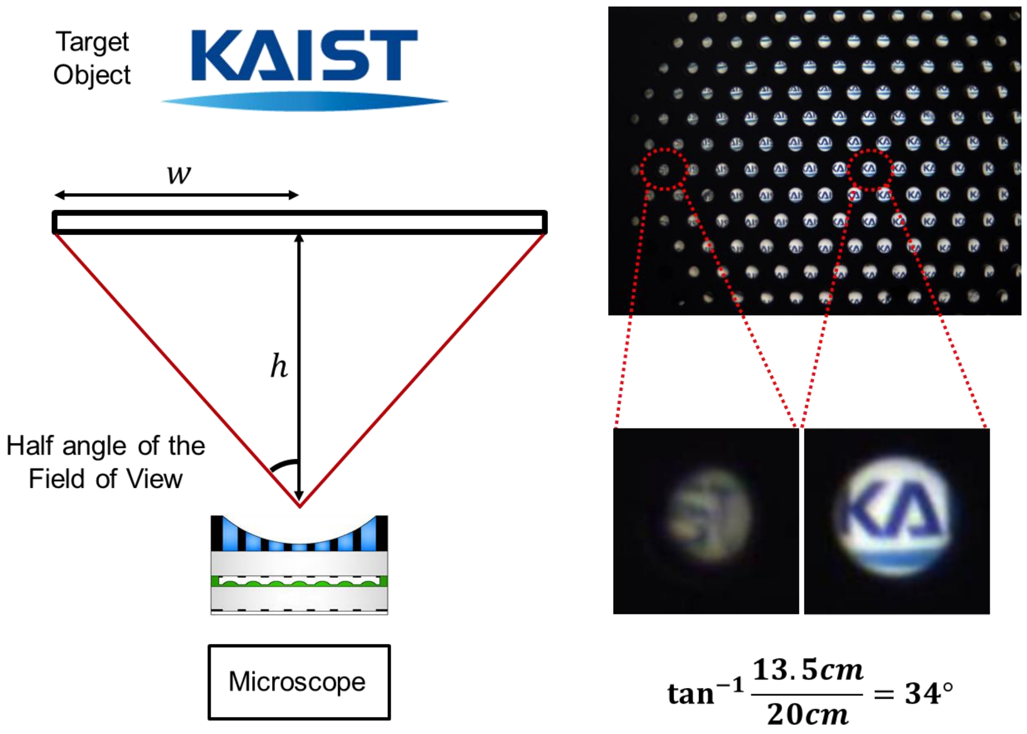

Supplementary Figure S3 | Field-of-view measurement of the camera. The FOV is trigonometrically calculated from the distance between the object and the camera and from the width of the captured object. The object distance is 20 cm and the half width of the imaged target picture is 13.5 cm, which indicates 68 degrees of the total FOV.
